# NOV/CCN3 attenuates inflammatory pain through regulation of matrix metalloproteinases-2 and -9

**DOI:** 10.1186/1742-2094-9-36

**Published:** 2012-02-21

**Authors:** Lara Kular, Cyril Rivat, Brigitte Lelongt, Claire Calmel, Maryvonne Laurent, Michel Pohl, Patrick Kitabgi, Stéphane Melik-Parsadaniantz, Cécile Martinerie

**Affiliations:** 1 INSERM UMR_S 938, Centre de Recherche de Saint-Antoine, Hôpital Saint-Antoine, Paris F-75012, France; 2 INSERM UMR_S 975; CNRS UMR 7225, Centre de Recherche de l'Institut du Cerveau et de la Moelle Epinière, Hôpital Pitié-Salpêtrière, Paris F-75013, France; 3 INSERM UMR_S 702, Hôpital Tenon, 4 rue de la Chine, 75970 Paris cedex 20, France; 4 Université Pierre et Marie Curie 6, Paris, France; 5 Department of Anesthesiology and Pain Medicine, Health Sciences RR415, 1959 NE Pacific Street, Seattle, WA, USA

**Keywords:** NOV/CCN3, Inflammatory pain, Neuroinflammation, IL-1beta, TNF-alpha, CCL2, MMP-2, MMP-9, Allodynia

## Abstract

**Background:**

Sustained neuroinflammation strongly contributes to the pathogenesis of pain. The clinical challenge of chronic pain relief led to the identification of molecules such as cytokines, chemokines and more recently matrix metalloproteinases (MMPs) as putative therapeutic targets. Evidence points to a founder member of the matricial CCN family, NOV/CCN3, as a modulator of these inflammatory mediators. We thus investigated the possible involvement of NOV in a preclinical model of persistent inflammatory pain.

**Methods:**

We used the complete Freund's adjuvant (CFA)-induced model of persistent inflammatory pain and cultured primary sensory neurons for *in vitro *experiments. The mRNA expression of NOV and pro-inflammatory factors were measured with real-time quantitative PCR, CCL2 protein expression was assessed using ELISA, MMP-2 and -9 activities using zymography. The effect of drugs on tactile allodynia was evaluated by the von Frey test.

**Results:**

NOV was expressed in neurons of both dorsal root ganglia (DRG) and dorsal horn of the spinal cord (DHSC). After intraplantar CFA injection, NOV levels were transiently and persistently down-regulated in the DRG and DHSC, respectively, occurring at the maintenance phase of pain (15 days). NOV-reduced expression was restored after treatment of CFA rats with dexamethasone. *In vitro*, results based on cultured DRG neurons showed that siRNA-mediated inhibition of NOV enhanced IL-1β- and TNF-α-induced MMP-2, MMP-9 and CCL2 expression whereas NOV addition inhibited TNF-α-induced MMP-9 expression through β_1 _integrin engagement. *In vivo*, the intrathecal delivery of MMP-9 inhibitor attenuated mechanical allodynia of CFA rats. Importantly, intrathecal administration of NOV siRNA specifically led to an up-regulation of MMP-9 in the DRG and MMP-2 in the DHSC concomitant with increased mechanical allodynia. Finally, NOV intrathecal treatment specifically abolished the induction of MMP-9 in the DRG and, MMP-9 and MMP-2 in the DHSC of CFA rats. This inhibitory effect on MMP is associated with reduced mechanical allodynia.

**Conclusions:**

This study identifies NOV as a new actor against inflammatory pain through regulation of MMPs thus uncovering NOV as an attractive candidate for therapeutic improvement in pain relief.

## Background

A growing body of evidence supports the fact that sustained neuroinflammation and particularly the release of pro-inflammatory cytokines and chemokines (including TNF-α, IL-1β, IL-6 and CCL2) strongly contributes to the pathogenesis of pain [1-5]. More recently, emerging studies have established that the extracellular matrix (ECM) components, particularly matrix metalloproteinases (MMPs) actively participate in the generation and maintenance of pain thus uncovering new targets for potential analgesics [6-11]. As a consequence, there is now a growing interest in the identification of factors that could modulate these families of molecules. Interestingly, NOV/CCN3, a founder member of the family of matricellular proteins called CCN (CYR61/CCN1, CTGF/CCN2, NOV/CCN3), has been involved in the regulation of cytokines, chemokines and MMP in several cell systems and therefore represents an attractive candidate for this role.

The nephroblastoma overexpressed gene (NOV) [12] encodes a secreted cysteine-enriched multimodular protein that acts as a localized multivalent signal integrator, primarily mediating its activities through interactions with specific dimers of integrins [13]. Previous findings have shown that NOV is highly expressed in the nervous system, especially in the spinal cord and in the dorsal root ganglion (DRG) during human and murine development [14-16]. This protein is also detected in the cerebrospinal fluid [17] and plays a role in the maturation of granular neuron precursors [18]. However, its roles in the adult central nervous system remain elusive. In addition, a relation between NOV and pro-inflammatory mediators has been reported in several cell systems. NOV differentially modulates the expression of MMP-1, -3, -2, -9 and -13 regarding its pro- or anti-motility effects in a cell-type manner [19-23]. Moreover, in non-nervous cell systems, NOV is regulated by cytokines (TGF-β, TNF-α and IL-1β) and is able to modulate their activities exerting anti-fibrotic and anti-inflammatory effects in a cell-type specific manner [24,25]. We have recently shown that in primary cultured astrocytes NOV expression is regulated by the cytokines TGF-β, TNF-α and IL-1β and that NOV specifically induces the expression of cytokines (IL-10) and chemokines (CCL2 and CXCL1) through distinct integrins and signaling mechanisms [25,26]. Altogether, these findings suggest a potential role for NOV in neuroinflammatory processes and led us to investigate the involvement of NOV in the development and persistence of complete Freund's adjuvant (CFA)-induced inflammatory pain. Here, in this preclinical model of inflammatory pain, we show that NOV may have an important function in limiting the deleterious effects of pro-inflammatory cytokines particularly on MMP-2 and MMP-9 expression in the nociceptive system, thereby exerting an anti-nociceptive effect.

## Methods

### Animals

Male Sprague-Dawley rats (Janvier), weighing 200 to 250 g (5 to 6 weeks of age) at the beginning of the experiments, were used. They were housed four per cage under standard conditions of light and temperature, for at least one week before and throughout the whole experimental period. Commercial chow pellets and tap water were available *ad libitum*. Animal-related procedures were approved by the French Ethics Committee in Animal Experiment (Comité d'Ethique pour l'Expérimentation Animale Charles Darwin, n°Ce5/2010/007) and carried out according to the French Standard Ethical Guidelines for Laboratory Animals.

### Chemicals and antibodies

Complete Freund's adjuvant, dexamethasone, collagenase, penicillin/streptomycin, transferrin, sodium selenite, putrescin, progesterone, corticosterone, triiodothyronine, insulin, cytosine arabinoside, proteases and phosphatases inhibitor cocktails, horseradish peroxidase-conjugated secondary antibodies and rat TNF-α and IL-1β cytokines were purchased from Sigma-Aldrich. MMP-9 inhibitor (Inhibitor-I) was purchased from Calbiochem. Lipofectamine RNAiMax and SiRNAs were obtained from Qiagen. F-12 nutrient mixture culture medium with L-glutamine, HBSS, trypsin, horse, goat and donkey sera and secondary antibodies conjugated to Alexa Fluor 488 or 594 were purchased from Invitrogen.

The primary antibodies used in this study were mouse anti-glial fibrillary acidic protein (GFAP) and anti-glyceraldehyde-3-phosphate dehydrogenase (GAPDH) from Chemicon, goat polyclonal anti-Iba1 (Abcam), mouse anti-neuronal nuclei (NeuN, Chemicon), mouse anti-neurofilament 200 (NF200, Sigma-Aldrich), fluorescein isolectin B4 (IB4, Vector) and guinea pig anti-calcitonin gene-related peptide (CGRP, Abcys SA, [27]). For NOV immunochemical detection and western blotting experiments, we used a homemade affinity-purified rabbit antibody (referred as CT-Mu, anti-mouse NOV antibody). This antibody was raised against a peptide specific to the carboxyl-terminal region of mouse NOV (amino acids 336 to 354, QNNEAFLQDLELKTSRGEI) which was coupled to activated keyhole limpet hemocyanin36 and used to generate a rabbit polyclonal antibody (CT-Mu). The CT-Mu immunoglobulin G (IgG) fraction was purified using standard methods on a CT-Mu peptide affinity column. The flow-through resulting from CT-Mu purification and containing IgG depleted from NOV specific IgG was used as a negative control [26]. Commercial human NOV protein was obtained from R&D Systems. Recombinant human NOV protein was produced using a baculovirus expression system in insect cells and purified as previously described [28].

### Drug treatments and experimental design

Inflammatory pain was induced by injecting subcutaneously an emulsion of 100 μl of CFA 50% dissolved in 9‰ NaCl (saline) into the left hind paw while the rats were under brief isoflurane anesthesia. Sham rats received a 100 μl injection of saline. For characterization of NOV expression in the CFA model, injured and corresponding sham animals (n = 4 rats/group) were sacrificed in the time course experiment at days 1, 3, 5, 7, 14, 21 and 60 after CFA injection. Two independent experiments were performed.

Water soluble dexamathesone (2 mg/kg i.p. dissolved in saline) was daily administered for 4 consecutive days (n = 4 rats/group) starting from day 9 post-CFA or saline injection. The control groups received four injections of saline in the same volume. The experiment was performed twice.

To proceed to small interfering RNA (SiRNA) intrathecal delivery, control non silencing SiRNA or specific NOV SiRNA (see below) were prepared immediately prior to administration by mixing the RNA solution (100 μM in annealing buffer) using two transfection reagents, either i-Fect™ in a ratio of 1:4 (w/v) (Neuromics, [29]) or Transductin™ complex respecting a molar ratio of 1:10 (IDT, [30]) following manufacturers protocols. At these ratios, the final concentration of RNA as an RNA/lipid or peptide complex was 2 μg in 10 μl. Ten microliters of NOV siRNA or control were delivered by intrathecal injection. Injection was given daily for 3 consecutive days starting from day 3 after CFA or vehicle injection (n = 3 to 4 rats/group) and tissues were harvested 24 h after the last injection for RNA analysis.

For behavioral and protein analyses, rats were daily injected intrathecally under brief isoflurane anesthesia with 2 μg of NOV siRNA (160 pmoles in i-Fect™ transfection reagent) or control siRNA on the day of CFA injection and then on the two following days (n = 8 rats/group). To test the NOV effect on pain intensity, three micrograms of commercial human NOV protein (in 12.5 μl saline) were delivered for 4 consecutive days, starting on the 9th day after CFA or saline injection. Control animals were injected intrathecally with the same volume of vehicle (n = 8 rats/group). Similarly, CFA rats were injected with 5 μg of MMP-9 inhibitor or vehicle (10% DMSO) from day 9 to day 12 post-CFA injection (n = 6 rats/group). Last intrathecal (i.t) injection was given 6 hours or 24 hours before animals were killed. Tissues were used for protein analysis.

### Behavioral experiments

After arrival in the laboratory, animals were left to accustom to the animal care unit for a week. During the seven days before the experiment, rats were weighed daily and placed in the test room for one hour. Mechanical allodynia (painful response to normally innocuous tactile stimuli) was evaluated the last two days preceding the scheduled experiment day (-d2 and -d1) as well as before the CFA-injection (d0) and each day of the experiment. Mechanical allodynia was measured using calibrated von Frey filaments (Bioseb) as described by Chaplan [31]. Animals were acclimated for 20 minutes in inverted individual cages on top of a wire mesh to provide access to the ventral side of the hind paws. The von Frey filaments were presented perpendicularly to the plantar surface of the selected hind paw, and then held in this position for approximately 5 seconds with enough force to cause a slight bend in the filament. A positive response was indicated by an abrupt withdrawal of the paw. A 50% paw-withdrawal threshold (PWT) was determined by increasing and decreasing the intensity and estimated using the Dixon's up-down method [32] that is, 10^(Xf + kΔ)^/10 000, where Xf = the value of the last von Frey filament employed, k = Dixon value for the p positive/negative pattern, and Δ = the logarithmic difference between stimuli. On treatment days, rats were tested prior to anesthesia followed by i.t administration of drugs. Data are presented as mean standardized values ± SEM and were analyzed by analysis of variance (ANOVA) followed by the Student-Newman-Keuls' post hoc test analysis.

### Sacrifice and tissue dissection

Rats were deeply anesthetized with pentobarbital sodium (150 mg/kg) and perfused transcardially with saline. The lumbar part of the spinal cord and DRG were removed and placed in a refrigerated cold plate (0 to 4°C). The spinal lumbar enlargement (L5-L6) was divided into left (lesioned side) and right parts by sagittal cut, and then into their dorsal and ventral ones by a horizontal cut passing through the ependymal canal. Dorsal quadrants of spinal cord and DRG were rapidly frozen in liquid nitrogen then kept at -80°C until being processed for RNA or protein analysis. Rats used for immunohistochemical study were perfused transcardially with heparin (1,000 U/ml) in saline followed by 500 ml of a freshly prepared solution of 4% paraformaldehyde (PAF) in PBS, pH 7.4. The dissected DRG and lumbar spinal cord were post-fixed overnight in 4% PAF at 4°C then cryoprotected in 15% sucrose (24 hours, 4°C) before being frozen at -40°C in isopentane.

### Immunohistochemistry

Fourteen- and twenty-micrometer cryostat sections of DRG and lumbar spinal cord, respectively, were cut with a cryostat (Leica CM3050S). Sections were incubated in a 1X PBS containing 0.1% Triton and 6% serum prior to incubation with primary antibodies. Primary antibodies used for this study were CT-Mu anti-NOV murine (1:5000 for spinal cord, 1:1,000 for DRG), anti-GFAP (1:800), anti-Iba1 (1:500) to detect glial markers, anti-NeuN (1:500), anti-NF200 (1:800), fluorescein isolectin B4 (IB4 1:500) and anti-CGRP (1:800) for neuron detection. Bound antibodies were detected using secondary antibodies conjugated to Alexa Fluor 488 or 594. A tyramide signal amplification was used to optimize NOV detection in spinal cord the following manufacturer's protocol (TSA Fluorescein System, PerkinElmer). Sections were finally mounted in the presence of DAPI to counterstain nuclei (Vector).

### Primary sensory neurons culture

Thoracic and lumbar DRG were removed from 3 young adult rats and were chemically and mechanically dissociated into single isolated neurons by enzyme treatment of 0.125% collagenase for 2 hours, followed by trypsin and DNAse for 10 minutes at 37°C and by trituration with Pasteur's pipette in F-12 medium. The cell suspension was then passed over a 70 μm filter, centrifuged and the pellet was resuspended in the F-12 nutrient mixture culture medium with L-glutamine containing 5% horse serum, 1% penicillin/streptomycin, glucose (4.5 g/l) and supplemented with 1.5% N3 medium (0.1% BSA, 10 mg/ml transferrin, 500 μg/ml insulin, 5.8 μM sodium selenite, 20 μM putrescin, 4 μM progesterone, 11 nM corticosterone, 3 μM triiodothyronine, in HBSS). Cells were plated on polylysine-coated wells (10 μg/ml). Cytosine arabinoside (1 μM) was added to eliminate mitotic cells including Schwann cells and fibroblasts. Cultures were maintained at 37°C in a water-saturated atmosphere with 5% CO_2 _for 5 days before the experiment. On the fifth day of culture, neurons exhibited globular cell bodies and extended axonal processes.

### Infection of primary sensory neurons culture with NOV adenovirus

Viral vectors used were either adenoviral vector containing the NOV gene under the control of a cytomegalovirus promoter (Ad-NOV, 1.1 × 10^11 ^infectious particles/ml) kindly provided by Dr C. Haberberger (Fibrogen Inc) or a control virus (Ad-null, 2.2 × 10^11 ^infectious particles/ml). Cells were infected with viruses at the ratio of 500 infectious particles/cell in culture medium containing 1% horse serum for 2 hours at 37°C. After 2 hours, media were replaced by serum deprived medium for an additional 48 hours prior to treatment. Neither the Ad-NOV nor the mock-infection showed deleterious cytotoxicity.

### SiRNA and *in vitro *transfections

Specific siRNA targeting NOV, integrins β_1_, β_3 _or β_5 _was used as previously described [26]. A siRNA with a non-silencing oligonucleotide sequence (control, Ctr) showing no known homology to mammalian genes was used as a negative control. Cultured DRG cells were transfected with siRNA (1.5 μg, 120 pmoles) using RNAiMax transfection reagent according to the manufacturer's instructions. Forty-eight hours after transfection, cultures were serum deprived and treated with cytokines and/or NOV for 6 to 24 hours. Cells were harvested and subjected to reverse transcription and real-time PCR. Conditioned media were collected for immunoblotting and detection of MMP-2 and -9 enzymatic activities.

### Real-time RT-PCR

Total RNA was extracted from DRG, dorsal horns of spinal cord or cultured DRG neurons using the Nucleospin RNA II kit (Macherey-Nagel). Then, first-strand cDNA was synthesized from 500 ng total RNA using the High Capacity cDNA Reverse Transcription kit (Applied Biosystems) according to the manufacturer's protocol. Quantitative real-time PCR amplifications were performed on the ABI 7300 apparatus (Applied Biosystems). The real time PCR amplification mixture (20 μl) contained 2 μl of 1/10 diluted cDNA template, 10 μl of Kit Power SYBR Green Master Mix (Applied Biosystems), 6 μl of nuclease-free water and 2 μl of primer mix (5 μM). Standard curves for target and housekeeping genes were generated by serial dilution. Specific primers (Sigma-Aldrich) used for amplification of target genes were designed using the online Primer3 Input program and verified for specificity on BLAST. Rat primers sequences are listed below. The comparative Ct method [33] was used to calculate gene expression values. The ribosomal S26 was used as a housekeeping gene 1.

**Table:** 

Genes	Sense primers	Antisense primers
NOV/ccn3	agtcatggtcattggaacctgta	ggacaactccttcactatgtttctt

CCL2	ccagaaaccagccaactctc	gctacaggcagcaactgtga

IL-6	ttccaatgctctcctaatgga	ggtttgccgagtagacctca

IL-1β	gcaatggtcgggacatagtt	gagacctgacttggcagagg

TNF-α	agtccgggcaggtctacttt	gggctctgaggagtagacga

Substance P (SP)	ggtccgacagtgaccaaatc	agttctgcattgcgcttctt

MMP-2	gccaaggtggaaatcagaga	atgtcagacaacccgagtcc

MMP-9	cgcaagcctctagagaccac	aaccacaggcttggatgtgt

S26	aaggagaaacaacggtcgtg	agagcttgggaagcacgtaa

### Substrate gel electrophoresis (zymography)

The gelatinases MMP-2 and MMP-9 proteolytic activities from rat tissues and from DRG neurons cultured in serum-deprived medium were detected by zymography. Conditioned media were centrifuged to remove cell debris and then stored at -20°C until further analysis. Frozen tissues were homogenized on ice by sonication in RIPA buffer. Twenty and thirty micrograms of total proteins (from conditioned media and DRG tissues, respectively) were subjected to electrophoresis under non-reducing conditions in 8% SDS-polyacrylamide gels copolymerized with 1 mg/ml gelatin. Gels were washed twice for 30 minutes in 2.5% Triton X-100 to remove SDS, incubated in substrate buffer (50 mM Tris-HCl, 5 mM CaCl2, 1 μM ZnCl2, 0.01% NaN3, pH 7.5) overnight at 37°C, stained in 0.5% Coomassie Blue G in 40% methanol, 10% acetic acid for 30 minutes at room temperature and destained in 40% ethanol, 1% acetic acid. Clear proteolytic zones indicated the presence of gelatinases at their respective molecular weights.

### CCL2 measurement

CCL2 concentrations in primary sensory neuron culture supernatants were quantified by using an ELISA test (OptEIA ™, BD Biosciences Pharmingen) following the manufacturer's instructions. At least two independent experiments were performed.

### Western blotting

Frozen tissues were homogenized on ice by sonication in lysis buffer (20 mM Tris-HCl (pH 7.5), 150 mM NaCl, 1 mM Na_2_EDTA, 1 mM EGTA, 1% Triton, 2.5 mM sodium pyrophosphate, 1 mM b-glycerophosphate) (Cell Signaling) supplemented with protease and phosphatase inhibitor cocktails. Protein concentration was determined using the Bradford assay (Bio-rad) with BSA as the standard. Proteins present in 20 μg of lysates were analyzed. For the detection of secreted NOV from primary sensory neuron cultures, protein samples from conditioned medium were collected after precipitation by deoxycholate/trichloracetic acid (DOC/TCA). Protein samples were subjected to electrophoresis in 10% reducing SDS-PAGE before being transferred to polyvinylidene difluouride membranes (Millipore) for immunological detection. The membrane was incubated with the CT-Mu mouse anti-NOV polyclonal antibody diluted to 1:500 for 1 hour at room temperature, followed by horseradish peroxidase-conjugated secondary antibodies. Immunoreactive proteins were detected by Enhanced chemiluminescence (Amersham, GE Healthcare).

### Statistical analysis

Data are presented as mean values ± SEM and were analyzed with student *t*-test (two groups only) or ANOVA followed by the Student-Newman-Keuls' post hoc test analysis. A value of *P *< 0.05 was considered statistically significant.

## Results

### NOV is expressed by neurons of the DRG and dorsal horn of the spinal cord (DHSC)

**Figure 1:**
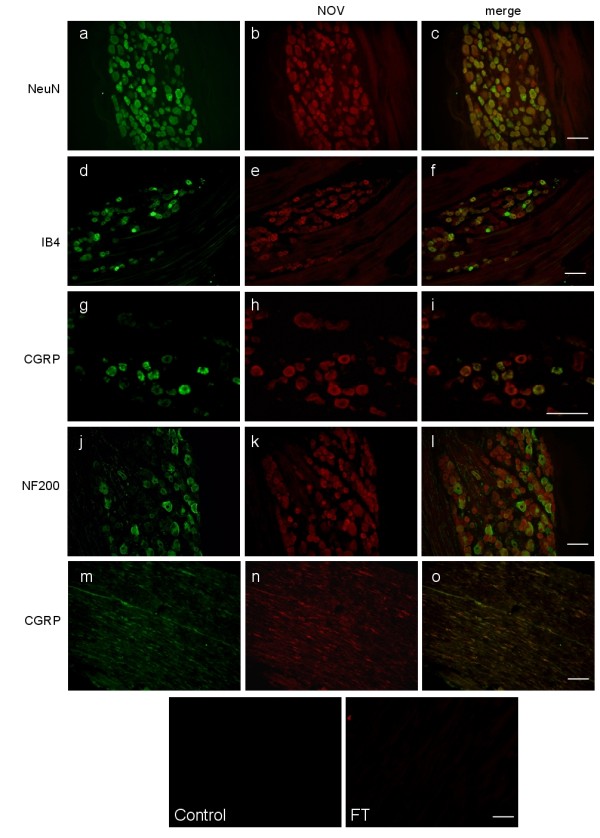
**Neuronal expression of NOV in DRG**. Immunolocalization using CT-Mu anti-mouse NOV antibody (red, b, e, h, k, n) and antibodies against neuronal markers NeuN (a), CGRP (g, m), IB4 (d) and NF200 (j). Overlay of the labeling (merge c, f, i, l, o). No primary antibody (control) and IgG depleted of NOV-specific IgG (flow through, FT) were used as negative controls. Scale: 100 μm.

NOV immunoreactivity (-IR) in nociceptive structures was first investigated by immunohistochemistry in the adult normal rat. In the DRG, NOV-IR was mostly neuronal as it displayed an overlapping pattern with the neuronal marker NeuN (Figure 1, a-c). NOV-IR was present in all neurons and was colocalized with both CGRP^+^-peptidergic and IB4^+^-non-peptidergic neurons (Figure 1, d-i) as well as with the large-diameter neuronal marker NF200 (Figure 1, j-l). NOV-IR could be detected in both neuronal cell bodies and processes (Figure 1, m-o).

**Figure 2:**
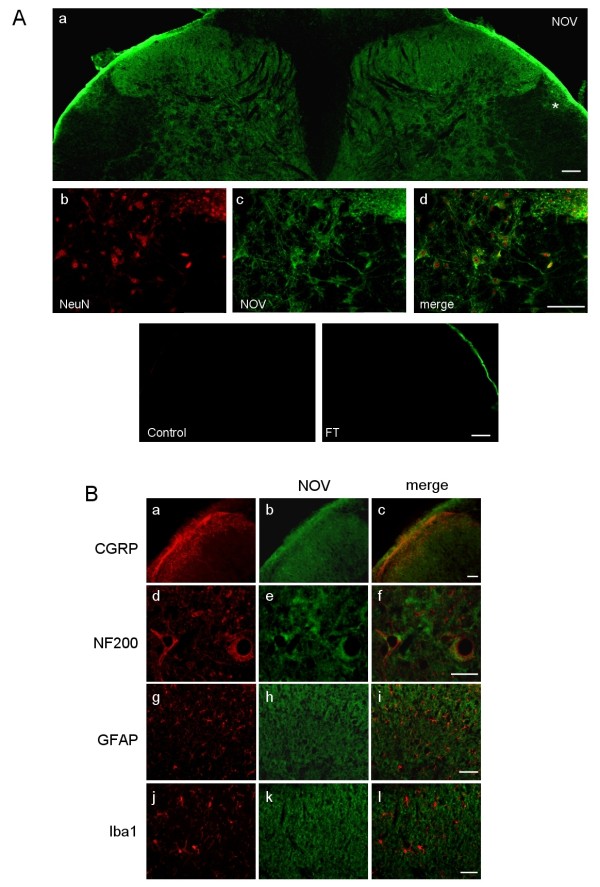
**Neuronal expression of NOV in the dorsal horn of the spinal cord**. (**A**) NOV immunolabeling in dorsal spinal cord *laminae *and in the dorsolateral funiculus (a, asterisk). (b-d) Immunolocalization using neuronal NeuN (b) and NOV (c) antibodies, overlay (merge, d). No primary antibody (control) and IgG depleted of NOV-specific IgG (flow through, FT) were used as negative controls. Scale: 100 μm. (**B**) Co-immunostaining using antibodies against NOV (green, b, e, h, k), neuronal (CGRP, a; NF200, d), astrocytic (GFAP, g) and microglial (iba1, j) markers. Overlay of the labeling (merge c, f, i, l). Scale: 50 μm.

In the lumbar DHSC, NOV-IR was detected in all lumbar levels and throughout all *laminae *(Figure 2A, a). NOV-IR was associated with the cytoplasm of spinal neurons of profound *laminae *and had a characteristic appearance in the ECM/pericytoplasmic region in the superficial *laminae *of the DHSC (Figure 2A, a-d). NOV-IR showed overlapping staining with CGRP and NF200 neuronal markers (Figure 2B, a-f). NOV-IR did not exhibit an overlapping staining with either the microglial marker Iba1 or the astrocytic marker GFAP (Figure 2B, g-l). Interestingly, a more moderate NOV-IR was also present in the dorsolateral funiculus of the spinal cord (Figure 2A, a asterisk). These data indicate that NOV is present in nociceptive structures and is essentially expressed by neurons in the DRG and DHSC.

### NOV expression is downregulated in DRG and DHSC at late stages of CFA-induced inflammatory pain

**Figure 3:**
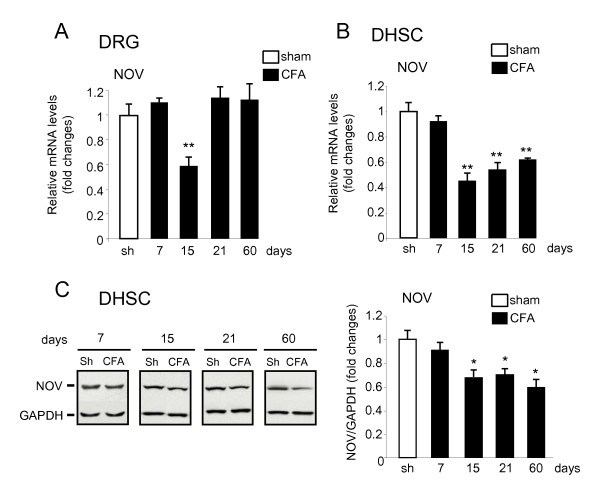
**NOV expression in DRG and dorsal horn of spinal cord (DHSC) during CFA-induced pain**. (**A, B**) NOV mRNA expression in DRG (**A**) and DHSC (**B**) ipsilateral to CFA injection in the hind paw. Transcript levels were quantified by RT-qPCR. Values were normalized to rat S26 mRNA levels. The corresponding control of the same time course (sham, sh) was set to 1. Data represent the mean value ± SEM of two independent experiments realized with four rats per condition. (**P < 0.01 CFA *versus *corresponding sham). (**C**) Expression of NOV protein in the DHSC. Representative western blot (left panel) and quantification of the protein levels normalized to GAPDH (right panel). Values are reported relative to the corresponding control (sham, sh) of the same time course (set to 1). Data represent the mean value ± SEM of two independent experiments realized with three rats per condition (*P < 0.05, CFA *versus *corresponding sham).

To identify whether NOV could play a role during the development of inflammatory pain, we first performed a time-course analysis of NOV expression in the DRG and DHSC after either CFA or saline hind paw injection. From day 1 to 7 after pain induction, no significant variation of NOV mRNA expression was observed in the ipsilateral DRG and DHSC (Figure 3A and 3B, and data not shown). By contrast, in the DRG, NOV mRNA levels were significantly decreased by 40% at day 15 (**P < 0.01, n = 6 to 8) and returned to their basal levels thereafter (Figure 3A). Spinal NOV mRNA expression was significantly downregulated by 58% starting from day 15 post-CFA (**P < 0.01, n = 8) and persisted at low levels for at least 60 days with a 40% decrease (**P < 0.01, n = 6) (Figure 3B). Consistent with the NOV mRNA expression pattern, NOV protein quantification on western blots in the ispilateral DHSC showed a significant (*P < 0.05, n = 5 to 6) decrease by 34%, 31% and 40% at 15, 21 and 60 days post-CFA, respectively (Figure 3C). No variation of NOV distribution was observed in this structure in CFA rats compared to respective shams (data not shown). These results reveal the fact that, in the time course of CFA-induced pain, NOV levels are downregulated in the ipsilateral DRG and DHSC, suggesting a potential functional association between reduced NOV levels at a late phase and the establishment of prolonged pain.

### Dexamethasone treatment of CFA rat restores NOV expression in nociceptive structures

**Figure 4:**
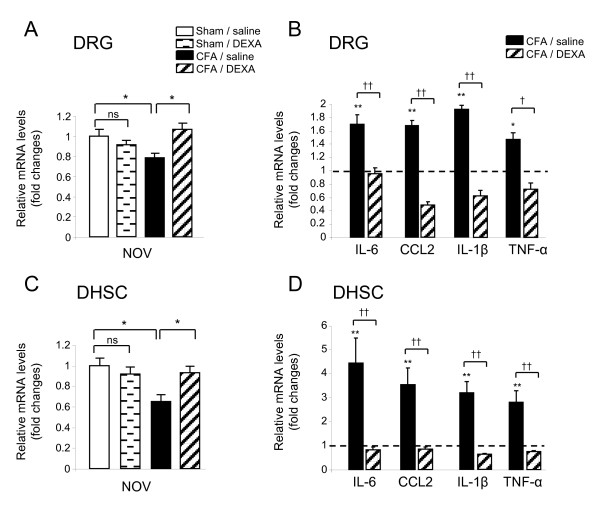
**Effect of dexamethasone on NOV expression in DRG and dorsal spinal cord**. mRNA expression of NOV (**A, C**) and the cytokines IL-6, CCL2, IL-1β and TNF-α (**B, D**) in the DRG (**A, B**) and DHSC (**C, D**) of rats treated with dexamethasone. Dexamethasone (DEXA, 2 mg/kg i.p) or vehicle (saline) were administered daily for 4 consecutive days to sham or CFA rats, starting from day 9 after CFA or saline injection into the paw. Transcript levels were quantified by RT-qPCR. Values were normalized to rat S26 mRNA levels and reported relative to the control group sham/saline set to 1 (dotted line in **B **and **D**). The data represent mean ± SEM of four rats per group of a representative experiment repeated twice (**A, C**: *P < 0.05, **P < 0.01 CFA/saline *versus *sham/saline or CFA-DEXA, **B, D**: *P < 0.05, **P < 0.01 CFA/saline *versus *sham/saline, †*P *< 0.05, ††*P *< 0.01 CFA/saline *versus *CFA/DEXA).

We then asked whether the treatment of CFA rats with the anti-inflammatory and analgesic glucocorticoid dexamethasone [34] could impact NOV expression. As shown in Figure 4A and 4C, 13 days after CFA-induced pain, NOV mRNA levels were significantly decreased in DRG and in DHSC (*P < 0.05, *n *= 4). Dexamethasone treatment re-established NOV-reduced expression in ipsilateral DRG and DHSC to levels measured in sham rats (*P < 0.05, n = 4). Dexamethasone had no significant effect on NOV expression in sham rats (Figure 4A and 4C). We also investigated mRNA expression of the cytokines IL-6, CCL2, IL-1β and TNF-α in saline and CFA rats ± dexamethasone. Cytokine expression was significantly upregulated in CFA rats, with a mean of 1.5-fold in the DRG and 3.5-fold in the DHSC compared to respective sham (Figure 4B and 4D, *P < 0.05, **P < 0.01, n = 4). Dexamethasone had an inhibitory effect on the induction of IL-6, CCL2, IL-1β and TNF-α in both DRG and DHSC (Figure 4B and 4D, †*P *< 0.05, ††*P *< 0.01, n = 4). This finding demonstrates for the first time *in vivo *a regulatory effect of dexamethasone on NOV expression and is in accordance with *ex vivo *studies [35]. This result is compatible with a possible involvement of NOV in inflammatory and/or nociceptive processes.

### Endogenous NOV regulates cytokine-induced nociceptive molecules in primary sensory neurons cultures

To gain insights into the possible role of NOV downregulation in the persistence of inflammatory pain, we carried out experiments based on cultured neurons from rat DRG. Because of the aforementioned effects of NOV on the regulation of cytokine, chemokine, and MMP expression in non-neuronal cell types, we explored the hypothesis that NOV was involved in such regulation in cultured DRG neurons. We first assessed the impact of siRNA-mediated NOV inhibition on the expression of MMP-9, CCL2, MMP-2 and substance P (SP) in response to cytokines stimulation.

**Figure 5:**
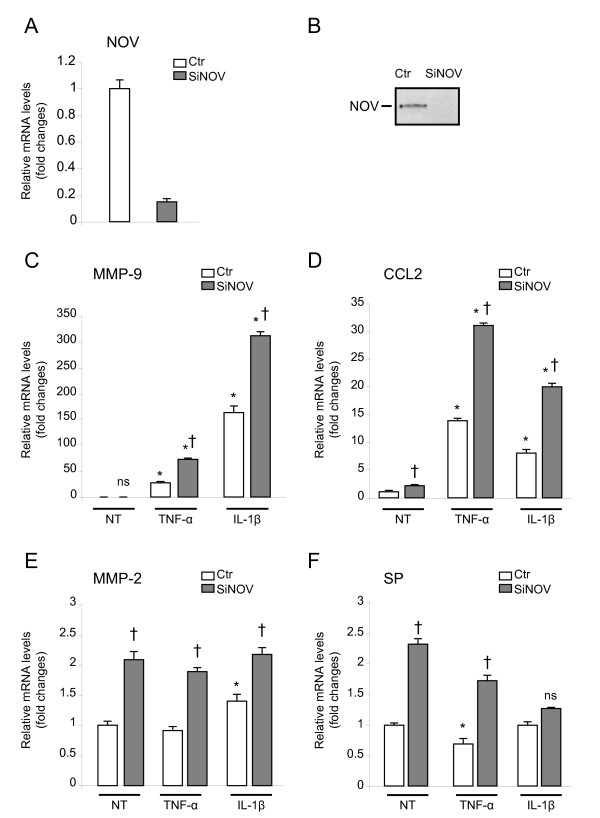
**Effect of NOV knockdown on cytokine-stimulated MMP-2/-9, CCL2 and SP mRNA in DRG neuronal cultures**. (**A, B**) NOV mRNA (**A**) and protein expression (**B**) 72 hours following transfection of primary sensory neurons with a control non-silencing siRNA (Ctr) or a specific siRNA against NOV (siNOV). NOV mRNA and secreted protein in the conditioned medium were quantified by RT-qPCR and western blot respectively. (**C-F**) Experiments were performed 48 hours following transfection with a control (control, Ctr) or a specific NOV siRNA (siNOV). MMP-9, CCL2, MMP-2 and substance P (SP) mRNA were evaluated by RT-qPCR after a 24 hour-exposure to cytokines TNF-α (1 ng/ml) or IL-1β (1 ng/ml). Data reported are fold changes *versus *the corresponding control of DRG cultures transfected with control siRNA exposed to normal medium (not treated, NT) and represent the mean of four values in a representative experiment performed at least two times with similar results (*P < 0.05 cytokine *versus *NT. †*P *< 0.05 siNOV *versus *Ctr).

DRG neuron cultures were transfected with a NOV-targeted siRNA (SiNOV) or a control siRNA (Ctr) containing a non-silencing sequence. SiNOV inhibited more than 80% of both NOV mRNA and protein expression at 72 hours (Figure 5A and 5B).

As shown in Figure 5C and 5D, NOV knockdown led to amplified stimulatory effects of the cytokines TNF-α and IL-1β on the mRNA levels of MMP-9 (siNOV × 73.9 ± 1.6 *versus *Ctr × 27 ± 2.2; siNOV × 312 ± 8.11 *versus *Ctr × 164 ± 11.5, respectively, †*P *< 0.05) and CCL2 (siNOV × 31 ± 0.7 *versus *Ctr × 14 ± 0.4; siNOV × 20 ± 0.75 *versus *Ctr × 8 ± 0.58, respectively, †*P *< 0.05). NOV depletion also impacted CCL2 expression under basal conditions (Figure 5D, not treated, NT, †*P *< 0.05).

As shown in Figure 5E and 5F, under basal condition (NT), MMP-2 and SP mRNA levels showed significant increases with a mean of 2-fold when NOV expression was abrogated (†*P *< 0.05). These increased MMP-2 and SP expressions were not amplified upon cytokine exposure (Figure 5E and 5F). Comparable results were obtained when DRG cultures were exposed to cytokines for 6 hours (data not shown).

**Figure 6:**
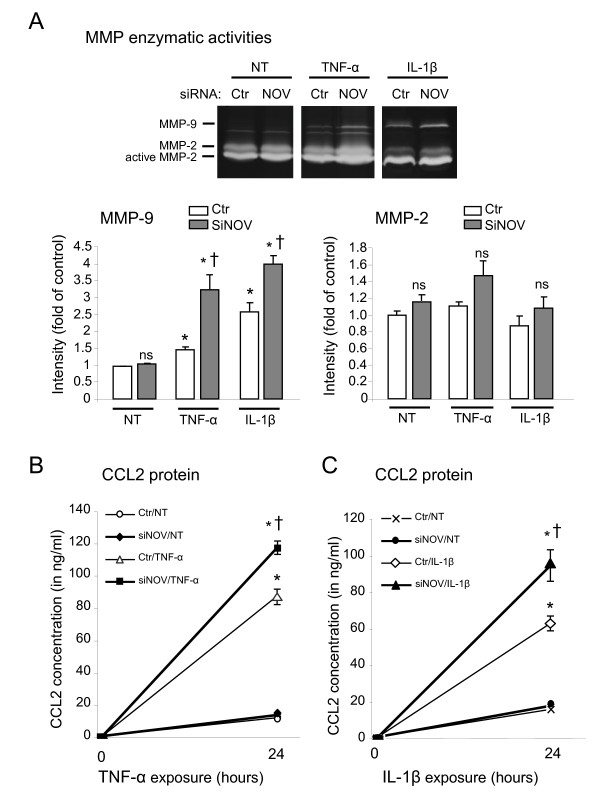
**Effect of NOV knockdown on cytokine-stimulated MMP-2/-9 activities and CCL2 concentration in DRG neuronal cultures**. Experiments were performed 48 hours following transfection of primary sensory neurons with a control non-silencing siRNA (Ctr) or a specific NOV siRNA (siNOV). (**A**) Upper panel, representative gelatin zymograph showing MMP-9 and MMP-2 enzymatic activities after transfection and treatment for 24 hours with cytokines TNF-α (1 ng/ml) or IL-1β (1 ng/ml). Lower panel, quantification of MMP-9 and MMP-2 gelatinolytic bands. Data represent the mean of four values ± SEM (ns, not significant, *P < 0.05 cytokine *versus *not treated (NT), †*P *< 0.05 siNOV *versus *Ctr). (**B, C**) CCL2 protein levels produced in conditioned medium of cultured DRG after transfection and exposure to cytokines TNF-α (1 ng/ml) (**B**) or IL-1β (1 ng/ml) (**C**) for 24 hours (or no treatment, NT) determined by ELISA. Data represent the mean of four values in a representative experiment performed at least two times with similar results (* *P *< 0.05 IL-1β or TNF-α *versus *NT. † *P *< 0.05 siNOV *versus *Ctr).

NOV depletion further enhanced cytokine-induced MMP-9 enzymatic activity with a mean of 1.8-fold (Figure 6A, †*P *< 0.05) and CCL2 protein levels with a mean of 1.6-fold (Figure 6B and 6C, †*P *< 0.05). NOV knockdown failed to significantly impact MMP-2 activity (Figure 6A).

Collectively, these data indicate that the blockade of endogenous NOV expression in cultured DRG neurons potentiates the induction of specific pro-nociceptive molecules by cytokines *in vitro*. Thus NOV deficiency leads to a greater response of sensory neurons to inflammatory stimuli thereby participating in the hypersensitization of nociceptors.

### NOV inhibits TNF-α-induced MMP-9 in DRG cultures through β_1 _integrin

**Figure 7:**
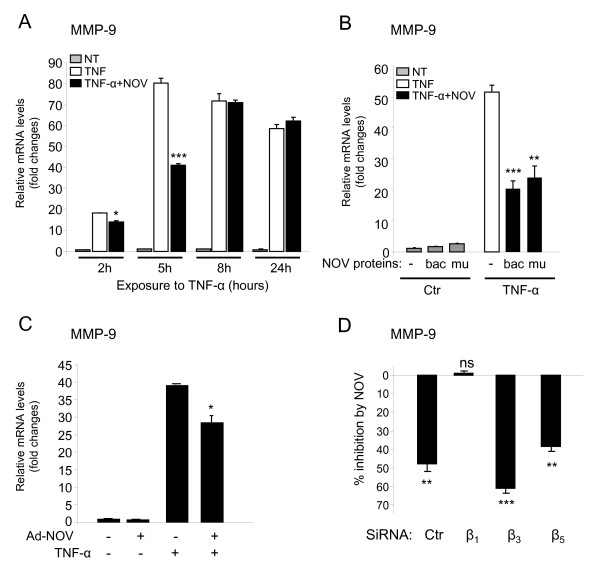
**Effect of NOV treatment on TNF-α-stimulated MMP-9 mRNA expression in DRG neuronal cultures**. (**A**) DRG cultures were exposed to TNF-α (1 ng/ml) for 1 hour prior to NOV treatment (1 μg/ml). Cells were harvested 2, 5, 8 or 24 hours after NOV treatment. (**B**) DRG cultures were exposed to TNF-α (1 ng/ml) for 1 hour prior to NOV treatment (1 μg/ml) for 5 hours with recombinant human NOV protein produced using a baculovirus expression system in insect cells (bac) or with commercial human NOV protein produced in a murine cell line (mu). (**C**) Cells were infected with NOV adenovirus (Ad-NOV) or control virus for 48 hours and then exposed to TNF-α (1 ng/ml) for an additional 12 hours. MMP-9 mRNA expression was evaluated by RT-qPCR. Data reported are fold changes *versus *the corresponding control of DRG cultures exposed to normal medium (NT, not treated) and represent the mean of four values in a representative experiment out of two experiments giving similar results (*P < 0.05, **P < 0.01, ***P < 0.001 TNF-α + NOV versu*s *TNF-α alone). (**D**) DRG cultures were exposed to TNF-α (1 ng/ml) for 1 hour prior to NOV treatment (1 μg/ml for 5 hours) 48 hours following transfection with a control non-silencing siRNA (Ctr) or a specific siRNA against integrin β_1_, β_3 _or β_5_. Values represent the percentage of inhibition ± SEM of MMP-9 by NOV upon TNF-α stimulation compared to TNF-α exposure of two independent experiments (ns: not significant, *P < 0.05, **P < 0.01, ***P < 0.001 TNF-α + NOV *versus *TNF-α).

We next focused our interest on MMP-9 modulation by NOV. We investigated MMP-9 mRNA levels in DRG neuron cultures exposed to TNF-α (1 ng/ml) for one hour prior to NOV addition (1 μg/ml) in time-course experiments. As shown in Figure 7A, addition of NOV decreased the stimulatory effect of TNF-α on MMP-9 mRNA expression. The NOV inhibitory effect on TNF-α-induced MMP-9 was rapid and transient since inhibition peaked at 5 hours with 50% inhibition (***P < 0.001) and declined thereafter. The specificity of this effect obtained with a home-purified recombinant protein (Figure 7B, bac, ***P < 0.001) was confirmed by using commercial human NOV produced in murine cell line (Figure 7B, mu, **P < 0.01). Similar results were obtained with NOV overexpression by infecting DRG cultures with an adenovirus recombinant for NOV (Ad-NOV, *P < 0.05) (Figure 7C). Thus, NOV counteracts the stimulatory action of TNF-α on MMP-9 expression in primary cultured sensory neurons.

So far, NOV has been shown to interact with the integrin dimers α_5_β_1_, α_6_β_1_, α_v_β_3 _and α_v_β_5 _and with Notch receptor [13] and, more recently, to modulate MMP-13 expression through α_v_β_3_/α_v_β_5 _engagement [23]. Thus, we wondered if NOV inhibitory action on cytokine-stimulated MMP-9 involved integrin receptors. Integrin subunits α_v _and β_1 _were expressed at the highest level (α_v _mRNA level was approximately 3-fold higher than α_5 _and α_6 _and, β_1 _was 50-fold more expressed than β_3 _and β_5_, data not shown). Thus, the integrins with which NOV interacts are expressed by cultured sensory neurons. As the β subunits in integrins are essential for signal transduction [36], we blocked β_1_, β_3 _or β_5 _expression using a siRNA approach. Cultured primary sensory neurons transfection with a β_1_-, β_3_- or β_5_-specific siRNA resulted in an 88%, 75% and 85% decrease of β_1_, β_3 _and β_5 _mRNA levels, respectively (data not shown). While β_3 _or β_5 _depletion did not prevent NOV from inhibiting the induction of MMP-9 by TNF-α (with respective 61% and 39% inhibitions, **P < 0.01, ***P < 0.001), β_1 _knockdown significantly abolished this inhibitory effect (Figure 7D). These data indicate that, in cultured primary sensory neurons, the NOV inhibitory action on TNF-α-induced MMP-9 expression requires interaction with integrin β_1_.

### MMP-2 and MMP-9 expression mirrors NOV expression in CFA-induced inflammatory pain and MMP-9 inhibition attenuates mechanical allodynia

**Figure 8:**
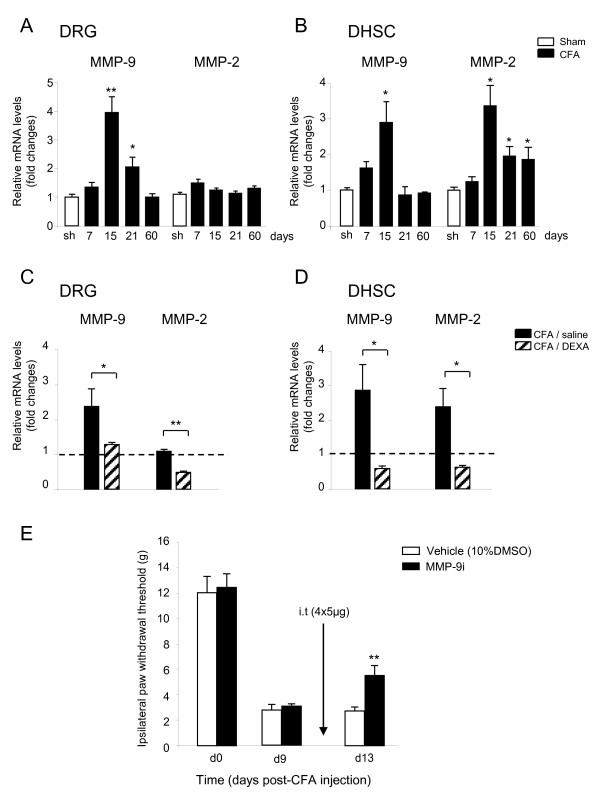
**MMP-2/-9 expression after CFA injection and the effect of MMP-9 inhibition on CFA-induced mechanical allodynia**. (**A, B**) Levels of MMP-9 and MMP-2 mRNA in the ipsilateral DRG (**A**) and DHSC, (**B**) after CFA or vehicle (sham) injection (*P < 0.05, **P < 0.01 CFA *versus *corresponding sham, n = 4). (**C, D**) Dexamethasone (DEXA, 2 mg/kg i.p) or vehicle (saline) were daily administered for 4 consecutive days to sham or CFA rats, starting from day 9 after CFA or saline injection into the paw. Transcript levels were quantified by RT-qPCR. Values were normalized to rat S26 mRNA levels and reported to the control group sham/saline set at 1 (dotted line). The data represent the mean ± SEM of 4 rats per group (*P < 0.05, **P < 0.01 CFA/saline *versus *CFA/DEXA). (E) MMP-9 inhibitor (MMP-9i, 5 μg) or vehicle (10% DMSO) were intrathecally (i.t) injected daily for 4 consecutive days starting from day 9 after CFA injection. The paw withdrawal threshold (g) was evaluated using the von Frey test. Data represent the mean ± SEM of six rats per group (**P < 0.01 MMP-9i- *versus *vehicle-treated rats).

It has been reported that following nerve injury, MMP-9 is transiently upregulated in injured DRG and participates in neuropathic pain induction, whereas MMP-2 displays a delayed increase in DRG and DHSC and is involved in a later phase of neuropathic pain maintenance [10]. So far, no characterization of MMPs expression in the CFA model of inflammatory pain has been carried out. Thus, we first examined MMP-2 and MMP-9 expression patterns in the DRG and DHSC during CFA-induced inflammatory pain. As shown in Figure 8A and 8B, MMP-9 mRNA levels were transiently upregulated in DRG on days 15 (×3.95 ± 0.55, **P < 0.01, n = 4) and 21 (×2 ± 0.36, *P < 0.05, n = 4) as well as in the DHSC 15 days (×2.8 ± 0.6, *P < 0.05, n = 4) after CFA injection, and returned to levels of sham rats thereafter. Markedly, MMP-2 expression showed no variation in DRG, but a mean 2-fold persistent upregulation in DHSC at late stages (×3.45 ± 0.66 on day 15; ×2 ± 0.66 on day 21 and × 1.94 ± 0.36 on day 60 post-CFA, *P < 0.05, n = 4). Moreover, dexamethasone treatment at late stages of inflammatory pain repressed MMP-2/-9 expression in DRG and DHSC of CFA rats (Figure 8C and 8D, *P < 0.05, **P < 0.01, n = 4). Thus, the induction of MMP-2 and MMP-9 mirrors reduced NOV expression at late stages in the establishment of inflammatory pain (Figure 3A and 3B).

In an acute model of inflammatory pain, carrageenan-induced mechanical allodynia is partially prevented in MMP-9-null mice [[10], suppl. data]. To test whether MMP-9 could impact CFA-induced persistent inflammatory pain, the mechanical allodynia of CFA rats treated with an MMP-9 inhibitor (MMP-9i) was evaluated using the von Frey filament test. As shown in Figure 8E, repeated intrathecal injections of MMP-9 inhibitor from day 9 to day 12 post-CFA injection significantly attenuated mechanical allodynia (5.5 ± 0.8 g in CFA rats treated with MMP-9i compared to 2.7 ± 0.3 g in CFA rats injected with vehicle, **P < 0.01, n = 6). Altogether, these data suggest a role for MMP-9 in the maintenance of CFA-induced inflammatory pain.

### Endogenous NOV knockdown upregulates MMP-2 and MMP-9 *in vivo *and increases mechanical allodynia of CFA rats

**Figure 9:**
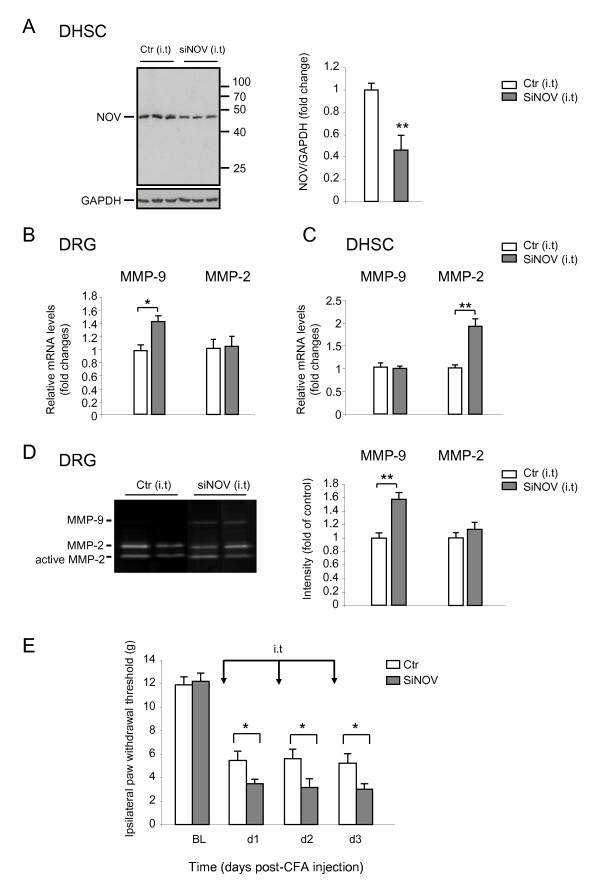
**Effect of in vivo endogenous NOV inhibition on MMP-2/-9 expression and mechanical allodynia**. In CFA rats, NOV-specific siRNA (2 μg) or control non-silencing siRNA (Ctr) were delivered intrathecally (i.t) daily for 3 consecutive days. (**A**) NOV protein levels in DHSC. Representative western blot (left panel) and quantification of protein levels normalized to GAPDH (right panel) (**P < 0.01, siNOV *versus *Ctr, n = 6) (**B, C**) Levels of MMP-9 and MMP-2 mRNA in DRG (B) and DHSC. (**C**) Transcript levels were quantified by RT-qPCR and values were normalized to rat S26 mRNA level. Data represent the mean value ± SEM of two independent experiments realized with three rats per condition (*P < 0.05 siNOV *versus *Ctr). (**D**) Representative gelatin zymograph showing MMP-9 and MMP-2 activities in DRG (left panel) and quantification of MMP-2 and MMP-9 gelatinolytic bands (right panel). Data represent the mean ± SEM of six rats per group (**P < 0.01 siNOV *versus *Ctr). (E) Paw withdrawal threshold (g) of CFA rats intrathecally injected with NOV-specific siRNA or control siRNA evaluated using the von Frey test. Data represent the mean ± SEM of eight rats per group (*P < 0.05 siNOV- *versus *Ctr-treated rats), BL: baseline.

We further investigated whether inhibition of NOV endogenous expression could modulate MMPs expression *in vivo*. As shown in Figure 9A, the intrathecal administration of NOV siRNA (siNOV) at early stages of CFA-induced pain (when neither NOV nor MMPs endogenous expressions are altered) resulted in a 40% inhibition of NOV mRNA expression in the DRG (data not shown) and a 58% reduction of NOV protein expression in the DHSC, compared with control siRNA-treated rats (**P < 0.01, n = 6). NOV downregulation led to a significant increase of MMP-9 mRNA levels in DRG of CFA rats (×1.43 ± 0.08, *P < 0.05, n = 6) without affecting MMP-2 expression (Figure 9B). Conversely, in DHSC, where MMP-9 mRNA levels showed no significant variation when NOV was inhibited, MMP-2 mRNA levels were significantly upregulated (×2 ± 0.17, **P < 0.01, n = 6) (Figure 9C). Consistent with NOV knockdown effects on MMPs mRNA in DRG, NOV siRNA delivery specifically increased MMP-9 proteolytic activity (x 1.59 ± 0.10 **P < 0.01, n = 6) and had no effect on MMP-2 activity in CFA rat DRG (Figure 9D). Due to lower MMP-2 and MMP-9 expressions in DHSC compared to DRG (5-fold and 2-fold less mRNA, respectively, data not shown), their gelatinase activities were below the detection threshold. Thus, in CFA-induced pain, NOV downregulation specifically affects MMP-9 and MMP-2 expressions in DRG and spinal cord, respectively.

In order to test whether endogenously produced NOV could modulate inflammatory pain, we evaluated the mechanical allodynia of CFA rats treated with NOV. As shown in Figure 9E, intrathecal delivery of siNOV resulted in a significant increase of mechanical allodynia compared to rats injected with control siRNA (*P < 0.05, n = 8). These data strongly suggest that endogenously produced NOV influences pain intensity and further support the hypothesis that NOV downregulation could participate in pain processes through upregulation of MMP-2 and MMP-9.

### NOV treatment of CFA rats inhibits MMPs and reduces mechanical allodynia

**Figure 10:**
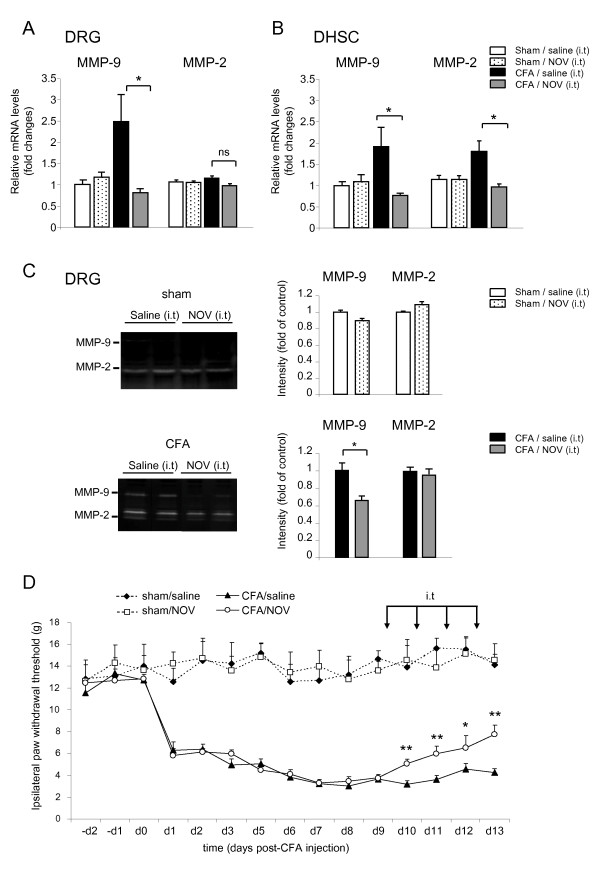
**Effect of NOV treatment of sham and CFA rats on MMP-2/-9 expression and mechanical allodynia**. NOV (3 μg/rat) was administrated daily by a single intrathecal injection (i.t) for 4 consecutive days starting from the 9th day after CFA injection. (**A, B**) Levels of MMP-2 and MMP-9 mRNA in the DRG (**A**) and DHSC (**B**) following intrathecal (i.t) treatment with NOV protein or vehicle (*P < 0.05 NOV- *versus *saline-treated CFA rats, n = 4 to 5). (**C**) Representative gelatin zymograph showing MMP-9 and MMP-2 activities (left panels) and quantification of MMP-2 and MMP-9 gelatinolytic bands (right panels). Data represent the mean ± SEM of three to four rats per group (*P < 0.05 NOV- *versus *saline-treated CFA rats). (**D**) The paw withdrawal threshold (g) was evaluated using the von Frey test. Data represent the mean ± SEM of eight rats per group (*P < 0.05, **P < 0.01 CFA/NOV- *versus *CFA/saline-treated rats).

We then wondered if NOV treatment of CFA rats at later stages had an impact on MMPs expression and nociceptive thresholds. To this aim, rats were intrathecally injected with NOV protein (3 μg) for 4 consecutive days starting from day 9 after CFA or saline injection. In accordance with MMPs pattern of expression in the CFA-induced pain model, as shown in Figure 10A and 10B, MMP-9 was upregulated both in the DRG and DHSC of CFA rats compared to sham animals, while MMP-2 was only significantly increased in DHSC. NOV treatment significantly abolished MMP-9 upregulation in ipsilateral DRG and DHSC (*P < 0.05, n = 4 to 5). Notably, NOV treatment resulted in significant inhibition of MMP-2 induction in DHSC (*P < 0.05, n = 4 to 5) and had no impact on its expression in DRG (Figure 10A and 10B). NOV administration to sham rats had no effect on MMPs expression. Consistent with NOV action on MMPs mRNA in DRG, NOV treatment specifically reduced MMP-9 proteolytic activity and had no effect on MMP-2 activity in CFA rats DRG (Figure 10C, *P < 0.05, n = 3 to 4). MMP-2 and -9 gelatinase activities were below the detection threshold in DHSC. Thus, *in vivo *NOV abolishes MMP-2 induction in the dorsal spinal cord and suppresses MMP-9 upregulation in both DRG and DHSC.

We tested whether concomitant with inhibition of MMPs, exogenous NOV could modulate persistent inflammatory pain. As shown in Figure 10D, CFA injection produced long-lasting mechanical stimulation hypersensitivity as reflected by the drastic decrease of withdrawal threshold to von Frey hairs dropping from 13 ± 0.5 g before CFA injection to 3.6 ± 0.3 g at day 9 post-CFA. Importantly, NOV treatment significantly attenuated the tactile allodynia developed in CFA rats (*P < 0.05, **P < 0.01, n = 8) and had no effect on basal pain perception in sham animals. These findings show NOV as an anti-allodynic molecule that is able to reduce established mechanical allodynia in the persistent phase of inflammatory pain and further suggest that NOV could exert its anti-allodynic effect through downregulation of MMP-2 and MMP-9.

## Discussion

Our study identifies NOV as a novel regulator of MMP-2 and MMP-9 expression in DRG and spinal cord during inflammatory pain processes and as an anti-allodynic molecule. To our knowledge, this is the first evidence for a function of NOV (or any members of the CCN family) in a pathophysiological inflammatory process implicating the nervous system and, particularly, pain pathogenesis.

We first explored the localization of NOV in nociceptive structures. Whereas NOV has been detected in neurons and astrocytes in the cortex [26], it was mostly expressed by neurons in the DRG and DHSC in the adult rat. Immunoreactivity was clearly present in a fibrillar pattern within DRG nerve axons and in the dorsolateral funiculus that contains major descending pathways, suggesting that spinal NOV could originate partly from its intrinsic expression in the spinal cord and from DRG and supraspinal structures axonal transport. These findings are in accordance with the expression pattern established previously in rat and human developing embryos [14-16] and represent the first description of NOV distribution within the DRG and DHSC in the adult rat.

We then wondered whether NOV was regulated during the establishment of prolonged inflammatory pain. Indeed, NOV was downregulated in DRG and DHSC in a time-dependent manner at a late phase of CFA-induced pain. Moreover, treatment of CFA rats with dexamethasone could restore NOV expression in both structures while decreasing the induced pro-inflammatory genes IL-6, CCL2, TNF-α, IL1-β, MMP-2 and MMP-9. These data further reinforce the hypothesis of a potential role for NOV in inflammatory and/or nociceptive processes.

We then investigated *in vitro *the possible involvement of NOV in neuroinflammatory processes. An extensive body of evidence has shown that the pro-inflammatory cytokines IL-1β and TNF-α, which, we confirmed, are upregulated at late times of CFA-induced pain, play critical roles in the generation and maintenance of pain [1,37]. Since NOV modulates the expression or activity of cytokines in several cell systems [25], we explored the involvement of NOV in such regulation in primary cultured sensory neurons. We found that inhibition of NOV endogenous expression enhances basal and cytokine-induced expression of the pro-nociceptive molecule CCL2. NOV action on CCL2 appears highly cell-type dependent since blockade of NOV in primary astrocytes leads to a reduced basal expression and release of CCL2 [26].

As previously reported, emerging studies point to the MMP family, especially the two gelatinases MMP-2 and MMP-9, as key upregulated pro-inflammatory mediators in a variety of CNS pathologies [38], including pain processes [8-11]. Herein, we present the first report of a stimulatory effect of NOV depletion on MMP-9. We also highlighted NOV as the first CCN member that inhibits TNF-α-induced MMP-9 expression in primary cultured sensory neurons. Such an inhibitory effect of NOV on MMP-9 expression is consistent with previous studies showing that NOV overexpression in Ewing's sarcoma and melanoma cells leads to repression of MMP-9 [21,22]. It appears that NOV, in concert with TNF-α, exerts differential effects depending on the cell-type. Indeed, NOV synergizes the inducer effect of TNF-α on CXCL1 expression in primary astrocytes or enables TNF-α to induce fibroblast apoptosis whereas it antagonizes the stimulatory action of TNF-α on the VCAM and ICAM adhesion molecules involved in endothelial inflammation [24,25,39,40]. The extent of NOV-mediated inhibition of MMP-9 expression in DRG neuron cultures upon TNF-α stimulation is similar to that reported on VCAM and ICAM in endothelial cells [24].

Recently, NOV has been shown to modulate MMP-13 through α_v_β_3_/α_v_β_5 _engagement and further activation of FAK, PI3K, Akt and NF-κB signaling pathways [23]. Here we demonstrate that in cultured sensory neurons, NOV regulation of cytokine-induced MMP-9 expression requires the engagement of integrin β_1 _subunit. Integrins mediate bidirectional transmembrane signaling between the ECM and intracellular second messengers [41]. Studies have shown that the engagement of integrin receptor β_1 _contributes to both neuropathic and chemically-induced pains [42-44], thus contrasting with our results. Interestingly, in neuronal cells, the ability of integrin β_1 _activation to favor or oppose cellular function depends both on the neuronal state and the precise molecular environment it encounters [45,46]. Moreover, the nature of the association between β_1 _and α integrin subunits plays an important role in pain hypersensitivity regarding nociceptive stimuli and specific second messenger cascades [47]. Thus, the effect of integrin β_1 _activation could have widely varying effects in cultured neurons. The intracellular mechanisms involved in NOV inhibitory action on cytokine-induced MMP-9 in this system remain to be elucidated.

While a pathophysiological role of MMP-2 and MMP-9 has been demonstrated in neuropathic pain, the relevance of these metalloproteinases in chronic inflammatory pain is still elusive. Our results show for the first time that inhibition of MMP-9 significantly decreases CFA-induced mechanical allodynia. Interestingly, intrathecal treatment with synthetic MMP-9 inhibitor has turned out to be more effective in reducing spinal nerve ligation-induced mechanical allodynia when continuously infused via an osmotic pump than when administered by repeated injections [10]. This suggests that the MMP-9 inhibitor partial effect on ipsilateral allodynia might be improved upon continuous delivery. Furthermore, since MMP-2 expression is increased in DHSC as well, it is plausible that pharmacological treatment of CFA rats with a combination of MMP-2 and MMP-9 inhibitors could exert a more potent anti-allodynic effect than when administered alone.

Next, we showed that NOV regulates MMP-2 and MMP-9 *in vivo *and that these regulations are associated with altered pain intensity. In CFA rats, the siRNA-mediated knockdown of NOV led to a specific upregulation of MMP-9 in the DRG and MMP-2 in the DHSC (and CCL2 in the DRG, data not shown), consistent with our *in vitro *data. Remarkably, endogenously produced NOV depletion resulted in increased tactile hypersensitivity. The compatible pattern of MMP-2/-9 and NOV expression at the late phase of inflammatory pain further raises the possibility that NOV reduction could specifically lead to transient MMP-9 induction in the DRG and a persistent increase of MMP-2 expression in the spinal cord, thereby contributing to the maintenance of pain. Admittedly, NOV is likely to act in concert with other specific partners of a given neuroinflammatory environment to modulate MMP-2/-9 expression. This is supported by our data showing NOV modulation of cytokine activities *in vitro *and cytokines induction at a late phase of inflammatory pain. In addition, NOV reduction at a late phase of the development of inflammatory pain contrasts with the early development of pain hypersensitivity indicating that NOV might not be involved in the early stage of pain induction but be required at a later precise time slot during pain pathogenesis. Even though the NOV-null mouse has been generated recently, its nervous system phenotype has not yet been reported. The NOV-null mouse shows normal development but enhanced neointimal hyperplasia in response to endothelial injury [48]. Our data thus reinforce the idea that NOV is highly important in tissue homeostasis especially in case of injury [24] and further highlight the interest of investigating pain symptoms in NOV-null mice. These findings indicate that endogenous NOV may have an important function in limiting the deleterious effects of pro-inflammatory cytokines particularly on MMPs expression in the nociceptive system.

Markedly, NOV intrathecal treatment abolished CFA-induced MMP-9 expression in both DRG and DHSC, whereas it abrogated MMP-2 induction in DHSC, concomitant with decreased CFA-induced ipsilateral allodynia. The NOV effect was partial but similar to that reported for drugs targeting blockade of NF-κB, JNK, IL-1β or TNF-α activities, which partially decrease pain hypersensitivity after CFA injection but fail to induce full recovery from pain symptoms [49,50]. Although the detailed molecular mechanisms involved in the NOV effect remain to be fully understood, these data reinforce the hypothesis that NOV exerts an anti-allodynic effect through modulation of MMP-2 and MMP-9. It is noteworthy that NOV treatment is able to reduce allodynia long after CFA administration, when pain symptoms remain robust. This is of clinical interest since studies have yielded debatable results revealing limited efficacies or even ineffectiveness of classical analgesics on mechanical hypersensitivity when administrated in post-CFA treatment [51-54]. More importantly, the cure of chronic inflammatory pain remains partial [55].

## Conclusions

Altogether, our findings reveal a role for NOV in the modulation of CFA-induced pain by exerting anti-inflammatory and anti-allodynic effects. Our study provides for the first time evidence that, besides their recently demonstrated roles in the pathogenesis of neuropathic pain, MMP-2/-9 could be involved in chronic inflammatory pain as well. We hypothesize that, during chronic inflammation, NOV downregulation in DRG and spinal cord contributes to MMP-2/-9 induction thereby participating in the maintenance of pain intensity. Because NOV appears as a novel mediator in the functional interplay between cytokine and MMPs in pain processes, it may alone or in concert with other drugs represent a new interesting analgesic. Knowing that recent evidence renders MMP-2/-9 useful targets for neuropathic pain relief [56], the investigation of NOV involvement in neuropathic pain would be worthwhile research, and preliminary data from our laboratory in this matter appear promising. Nevertheless further comprehensive research is needed to investigate the precise mechanisms involved in such function in order to improve therapeutic solutions.

## Abbreviations

CFA: complete Freund's adjuvant; CCN: cysteine rich 61/connective tissue growth factor/nephroblastoma overexpressed; DRG: dorsal root ganglia; DHSC: dorsal horn of the spinal cord; ECM: extracellular matrix; IL-1β: interleukin-1β; MMP: matrix metalloproteinase; NOV: nephroblastoma overexpressed; TNF- α: tumor necrosis factor-α.

## Competing interests

The authors declare that they have no competing interests.

## Authors' contributions

CM and SMP conceived, designed and supervised the study; LK participated in designing experiments, conducted experiments and prepared the manuscript. CR, BL, CC, ML and MP performed experiments and revised the manuscript. PK participated in drafting and revising the manuscript. All authors read and approved the final manuscript.
